# Detection of patients at high risk for nonocclusive mesenteric ischemia after cardiovascular surgery

**DOI:** 10.1186/s13019-018-0807-5

**Published:** 2018-11-16

**Authors:** Hiroshi Sato, Masanori Nakamura, Takeshi Uzuka, Mayo Kondo

**Affiliations:** 1 Department of Cardiovascular Surgery, Sapporo Medical University School of Medicine, S1W16, Chuo-ku, Sapporo, 060-8543 Japan; 2 Department of Cardiovascular Surgery, Sapporo City General Hospital, N11W13, Chuo-ku, Sapporo, 060-8604 Japan

**Keywords:** Nonocclusive mesenteric ischemia, Cardiovascular surgery, Risk model

## Abstract

**Objectives:**

Nonocclusive mesenteric ischemia (NOMI) is a rare but life-threatening complication after cardiovascular surgery. Early diagnosis and treatment is essential for a chance to cure. The aim of this study is to identify the independent risk factors for NOMI based on the evaluation of 12 cases of NOMI after cardiovascular surgery.

**Methods:**

We retrospectively analyzed 12 patients with NOMI and 674 other patients without NOMI who underwent cardiovascular surgery in our hospital. We reviewed the clinical data on NOMI patients, including their characteristics and the clinical course. In addition, we performed a statistical comparison of each factor from both NOMI and non-NOMI groups to identify the independent risk factors for NOMI.

**Results:**

The median duration between the cardiac surgery and the diagnosis of NOMI was 14.0 (10.3–20.3) days. The in-hospital mortality of NOMI patients was 75.0%. Age (*p* < 0.05), peripheral arterial disease (*p* < 0.001), postoperative hemodialysis (*p* < 0.001), intraaortic balloon pump (*p* < 0.05), norepinephrine (NOE) > 0.10γ (*p* < 0.0001), percutaneous cardiopulmonary support (*p* < 0.001), sepsis (*p* < 0.05), loss of sinus rhythm (*p* < 0.05), prolonged ventilation (*p* < 0.0001), and resternotomy for bleeding (*p* < 0.05) showed significant differences between NOMI and non-NOMI groups. In the multivariate logistic regression model, prolonged ventilation [odds ratio (OR) = 18.1, *p* < 0.001] and NOE > 0.10 μg/kg/min (OR = 130.0, *p* < 0.0001) were detected as independent risk factors for NOMI.

**Conclusions:**

We have identified the risk factors for NOMI based on the evaluation of the 12 cases of NOMI after cardiovascular surgery. This result may be useful in predicting NOMI, which is considered difficult in clinical practice. For the patient with suspected of NOMI who has these risk factors, early CT scan and surgical exploration should be performed without delay.

## Introduction

Nonocclusive mesenteric ischemia (NOMI) is a rare complication after cardiovascular surgery, and its incidence rates were reported to be about 0.4 to 9.0% [1–4]. Although the exact pathophysiology is currently unclear, it is assumed that the vasospasm of mesenteric artery results from the low perfusion during cardiopulmonary bypass (CPB) or the various intra/postoperative therapeutic medications [5]. NOMI is a serious complication with a reported 30 to 90% mortality rate [2, 4, 5]. Clinical signs, such as abdominal pain, vomiting, and hematochezia, can be seen but are not very specific. Also, the abnormal elevation of laboratory data has low specificity. The clinical sign is likely masked because the patient is often sedated and ventilated at the onset of NOMI; therefore, the diagnosis of NOMI is frequently difficult and delayed. In computed tomography (CT) scan findings, absence of bowel wall enhancement, pneumatosis intestinalis, and portal venous gas are specific radiological signs but do not necessarily appear in ischemic conditions. Therefore, constant monitoring of the possibility of intestinal ischemia for the high-risk patient and surgical exploration without delay are the only ways for the improvement of survival rate. [6–8]

However, there are few studies that have detected the risk factor of NOMI after cardiovascular surgery [2, 5]. In this study, we reviewed the clinical data of 12 cases of NOMI after cardiovascular surgery in our hospital. Then, we investigated each factor between the two groups and detected the independent risk factors for NOMI.

## Materials and methods

### Patients

From March 1, 2010 to December 31, 2018, among the patients who underwent cardiovascular surgery in our institution, we conducted a retrospective case-control study for 12 (1.74%) patients who developed NOMI after surgery and 674 (98.3%) other patients. We also included emergent surgical cases and off-pump cases. Excluded cases were the patients with type A aortic dissection who had mesenteric ischemia before surgery, thoracic endovascular aortic repair, and pericardial fenestration.

### Diagnosis of NOMI

**Fig. 1:**
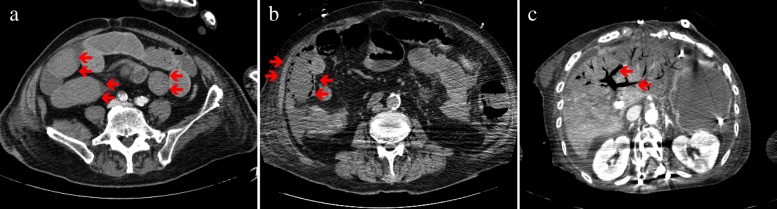
Red arrows indicate the CT findings of the diagnosis of NOMI in each images: **a** absence of bowel wall enhancement, **b** pneumatosis intestinalis, and **c** portal venous gas

After cardiovascular surgery, the patients with suspected NOMI because of abdominal distension with absence of bowel sounds, acute abdominal pain, vomiting, hematochezia, or abnormal laboratory data underwent urgent abdominal CT scans. NOMI was diagnosed by the confirmation of the presence of ischemic bowel signs (absence of bowel wall enhancement, pneumatosis intestinalis, or portal venous gas; (Fig. 1) without the occlusion or thrombus of the superior mesenteric artery in the CT scan findings and undisputed mesenteric ischemia by surgical exploration. All radiological signs in the CT scans were reviewed by the radiologist. The CT scan and surgical laparotomy were performed by the judgment of each operator to confirm the diagnosis.

### Definition of each analyzed data

Valve surgery included aortic valve replacement /aortic valve plasty, mitral valve replacement (MVR)/mitral valve plasty (MVP), and tricuspid annulus plasty. Thoracic aortic surgery included ascending aortic replacement (AAR)/total arch replacement (TAR)/hemi-arch replacement, and aortic root replacement/remodeling. Each analyzed factor was defined using the following criteria. Norepinephrine (NOE) > 0.10 μg/kg/min was defined as using NOE more than 0.10 μg/kg/min for more than 1 h after surgery. Low output syndrome (LOS) was defined as using mechanical support, that is, intraaortic balloon pump (IABP) support and percutaneous cardiopulmonary support (PCPS). Prolonged ventilation was defined as mechanical ventilation time after surgery of more than 24 h. Loss of sinus rhythm was defined as the documented loss of sinus rhythm for at least 6 h after surgery. Hyperlactatemia was defined as having a serum lactate level of > 5.0 mmol/L.

### Statistical analysis

Statistical analysis was performed using Mann-Whitney *U* test for continuous variables and χ^2^ test and Fisher’s exact test for categorical variables. Variables found associated with *P* < 0.05 in the univariate analysis were entered into a multivariate logistic regression analysis, using the stepwise selection method, to identify the factors independently associated with a definite risk factor of NOMI. All results were expressed as median (interquartile range). Statistical significance was set at *P* < 0.05 (two-sided). All data analyses were performed using the statistical program R version 3.2.1 (R Foundation for Statistical Computing, http://www.r-project.org/).

## Results

**Fig. 2:**
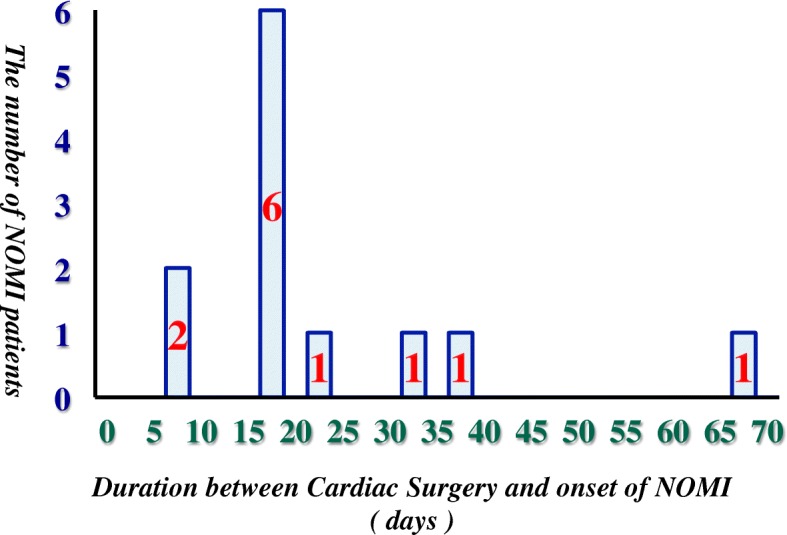
Relationship of the number of NOMI patients and duration between cardiovascular surgery and onset of NOMI

Among patients who underwent cardiovascular surgery in our institution, 12 (1.74%) cases developed NOMI. The background of NOMI cases, including patient characteristics, surgery type, and state at the onset of NOMI, is displayed in Table 1. The mean age of patients was 74.67 ± 7.6 years, and 8 (66.7%) patients were male and 4 (33.3%) patients were female. The details of the cardiovascular surgery were 5 (41.7%) off-pump coronary artery bypass graft surgery (OPCABG), 4 (33.3%) CABG, 2 (16.7%) valve surgery, and 2 (16.7%) thoracic aortic surgery. As initial symptoms and findings of NOMI, abdominal pain, vomiting, fever, hematochezia, hypotension, or hyperlactatemia were noted. At the onset of NOMI, 8 of 12 cases were under sedation due to mechanical ventilation. Nine (75.0%) cases died in the hospital and 3 (25.0%) survived. The median duration between the cardiovascular surgery and the onset of NOMI was 14.0 (10.3–20.3) days, and the onset of NOMI for 6 of 12 cases (50.0%) was between 15 and 20 days (Fig. 2).

**Table 1:** Background of 12 NOMI patients

No	Age	Sex	Surgery	Emergency	Initial symptoms/findings	Duration between Surgery and NOMI(days)	Patient state at the onset of NOMI					Result
							NOE > 0.10 μg/kg/min	LOS	Sepsis	HD	Sedation	
1	83	Male	TAR	No	Hyperlactatemia	13	Yes	No	No	Yes	Yes	Death
2	81	Male	OPCABG	No	Hematochezia	64	No	No	Yes	Yes	Yes	Death
3	67	Male	OPCABG	No	Abdominal pain	13	No	No	No	No	No	Survival
4	66	Male	OPCABG	No	Abdominal pain	34	No	No	Yes	No	No	Survival
5	77	Female	AAR	Yes	Hyperlactatemia	17	Yes	Yes	No	Yes	Yes	Death
6	81	Male	CABG	No	Hyperlactatemia	30	Yes	Yes	No	No	No	Death
7	86	Male	OPCABG	Yes	Hyperlactetamia	8	Yes	Yes	No	Yes	Yes	Death
8	69	Female	OPCABG	No	Hyperlactetamia	11	Yes	No	No	Yes	Yes	Death
9	67	Female	CABG	No	Hyperlactatemia	2	Yes	No	No	Yes	Yes	Death
10	76	Female	MVP, TAP	No	Vomiting	15	No	No	No	No	No	Survival
11	79	Male	CABG, MVR	No	Hypotension	15	Yes	Yes	Yes	Yes	Yes	Death
12	64	Male	CABG	Yes	Hyperlactatemia	5	No	Yes	No	Yes	Yes	Death

*NOMI* non-occlusive mesenteric ischemia, *NOE* norepinephrine, *LOS* low output syndrome, *HD* hemodialysis, *TAR* Total Arch Aortic Replacement, *OPCABG* off-pump coronary artery bypass graft surgery, *AAR* Ascending Aortic Replacement, *CABG* coronary artery bypass graft surgery, *MVP* mitral valve plasty, *TAP* tricuspid annuloplasty, *MVR* mitral valve replacement

NOE > 0.10 μg/kg/min was used for 7 of 12 cases within 24 h before the onset of NOMI. Cases 5 to 7 and 11 were in a state of LOS; furthermore, case 11 also had sepsis and underwent hemodialysis. Cases 5 and 7 underwent hemodialysis too, and Case 6 did not. Cases 1, 8, and 9 were not at a state of LOS and had sepsis but underwent hemodialysis while using NOE. NOE was not used for Cases 2 to 4, 10, and 12. Cases 2 and 4 had sepsis, and Case 12 was at a state of LOS and underwent hemodialysis. Cases 3 and 10 were not in any condition. As initial findings/symptoms, 7 of 12 cases showed hyperlactatemia. In others, 1 hypotension, 2 abdominal pain, 1 vomiting, and 1 hematochezia were found.

Between NOMI and non-NOMI groups, pre/intra/postoperative factors were summarized and subjected to statistical comparison (Tables 2 and 3). There was a significant difference in 10 factors: age (*p* < 0.05), peripheral arterial disease (PAD; *p* < 0.001), postoperative hemodialysis (*p* < 0.001), IABP (*p* < 0.01), NOE > 0.10 μg/kg/min (*p* < 0.0001), PCPS (*p* < 0.0001), sepsis (*p* < 0.01), loss of sinus rhythm (*p* < 0.01), prolonged ventilation (*p* < 0.0001), and resternotomy for bleeding (*p* < 0.01). Finally, these 10 factors were introduced for the multivariate logistic regression model as covariates and adjusted odds ratio (OR) were calculated. As a result, prolonged ventilation (OR = 18.1, *p* < 0.001) and NOE > 0.10 μg/kg/min (OR = 130.0, *p* < 0.0001) were detected as independent risk factors for NOMI (Table 4).

**Table 2:** Preoperative characteristics

Variables	NOMI (*n* = 12)	Non-NOMI (*n* = 674)	*P* value
Age, median (IQR) (years)	67 (64–74.5)	63 (29–70)	< 0.05
Age > 75, n (%)	7 (58.3)	193 (28.1)	< 0.05
Sex: Male, n (%)	8 (66.7)	430 (62.7)	0.99
COPD, n (%)	2 (16.7)	138 (20.4)	0.95
Diabetes, n (%)	5 (41.7)	248 (36.9)	0.77
Hemodialysis, n (%)	4 (33.3)	115 (16.8)	0.138
Hypertension, n (%)	8 (66.7)	421 (61.4)	0.775
Peripheral Arterial Disease, n (%)	5 (41.7)	43 (6.3)	< 0.001
Stroke, n (%)	1 (8.3)	20 (2.9)	0.314

*IQR* interquartile range, *COPD* chronic obstructive pulmonary disease

**Table 3:** Operative and postoperative characteristics and in-hospital mortality

Variables	NOMI (*n* = 12)	Non-NOMI (*n* = 674)	*P* value
Operative			
Operation types			
OPCABG, n (%)	5 (41.7)	136 (19.8)	0.078
CABG, n (%)	4 (33.3)	180 (26.2)	0.742
Valve surgery, n (%)	2 (16.7)	251 (36.6)	0.227
Thoracic Aortic, n (%)	2 (16.7)	121 (17.6)	0.95
Others, n (%)	0 (0)	37 (5.4)	0.95
Emergency, n (%)	3 (25.0)	77 (11.2)	0.155
CPB, n (%)	7 (58.3)	425 (62.0)	0.768
Operation time, median (IQR) (min)	422.8 (255–480)	340 (40–420)	0.139
ACC time, median (IQR) (min)	139 (112–140)	120.8 (28–160)	0.951
CPB time, median (IQR) (min)	195.5 (158–240)	188.8 (50–239)	0.889
Postoperative			
Hemodialysis, n (%)	8 (66.7)	123 (17.9)	< 0.001
IABP, n (%)	5 (41.7)	71 (10.3)	< 0.05
Loss of sinus rhythm, n (%)	9 (75.0)	201 (29.3)	< 0.05
Sepsis, n (%)	2 (16.7)	13 (1.9)	< 0.05
NOE > 0.10 μg/kg/min, n (%)	9 (75.0)	17 (2.5)	< 0.0001
PCPS, n (%)	4 (33.3)	20 (2.9)	< 0.001
Prolonged ventilation, n (%)	9 (75.0)	108 (15.7)	< 0.0001
Resternotomy for bleeding, n (%)	3 (25.0)	33 (4.8)	< 0.05
In-hospital mortality, %	75	5.4	< 0.0001

*IQR* interquartile range, *OPCABG* off-pump coronary artery graft bypass surgery, *CABG* coronary artery graft bypass surgery, *ACC* aortic cross-clamp, *CPB* cardiopulmonary bypass, *IABP* intra-aortic balloon pump, *NOE* Norepinephrine, *PCPS* percutaneous cardiopulmonary support

**Table 4:** Univariate and multivariate logistic regression model

Risk factor	Univariate analysis			Multivariate analysis		
	OR	95% CI	*P* Value	Adjusted OR	95% CI	*P* Value
Age > 75	3.52	1.10–11.20	< 0.05			
Peripheral Arterial Disease	10.30	3.12–33.80	< 0.001			
Postoperative hemodialysis	8.88	2.63–30.00	< 0.001			
IABP	5.65	1.75–18.30	< 0.01			
Loss of sinus rhythm	6.92	1.85–25.80	< 0.01			
Sepsis	10.10	1.98–50.90	< 0.05			
NOE > 0.10 μg/kg/min	52.40	15.00–183.00	< 0.0001	130.0	21.4–921.0	< 0.0001
PCPS	15.70	4.34–56.60	< 0.001			
Prolonged ventilation	15.10	4.02–56.70	< 0.0001	18.1	6.64–388.0	< 0.001
Resternotomy for bleeding	7.55	1.93–29.60	< 0.05			

Further analysis for these two variables showing significant difference in the multivariate logistic model has proceeded. Among the total 26 cases corresponding to NOE > 0.10 μg/kg/min, total quantity (μg/kg), duration (h), and maximum dose of NOE (μg/kg/min) were statistically compared between NOMI (9 cases) and non-NOMI (17 cases) groups. Similarly, among the total 117 cases corresponding to prolonged ventilation, maximum PEEP (cmH_2_O), total ventilation time (h), and index calculated from PEEP * ventilation time/body weight (BW) (cmH_2_O * h/kg) were statistical compared between NOMI (9 cases) and non-NOMI (108 cases) groups. As a result, all variables were higher in the NOMI group, but no significant difference was found between the two groups **(**Table 5**)**.

**Table 5:** NOE and ventilation factors of NOMI and non-NOMI groups

Variables	NOMI (*n* = 9)	Non-NOMI (*n* = 17)	*P* value
NOE factor			
Quantity, median (IQR) (μg/kg)	1237.2 (853.6–1850.1)	559.6 (331–2821.7)	0.403
Maximum dose, median (IQR) (μg/kg/min)	0.43 (0.28–0.75)	0.37 (0.21–0.56)	0.219
Duration, median (IQR) (h)	106.3 (38–146)	81 (37.3–208.3)	0.9
Variables	NOMI (n = 9)	Non-NOMI (*n* = 108)	*P* value
Ventilation factor			
Maximum PEEP, median (IQR) (cmH2O)	10 (10–14)	10 (8–10)	0.157
Total ventilation time, median (IQR) (h)	226 (116–308)	87 (51.5–189.5)	0.077
PEEP * ventilation time/BW index, median (IQR) (cmH_2_O * h/kg)	24.2 (15.6–36.9)	11.7 (6.5–28.7)	0.051

*IQR* interquartile range, *NOE* Norepinephrine, *PEEP* positive end expiratory pressure, *BW* body weight

## Discussion

NOMI is a rare but life-threatening complication after cardiovascular surgery. Although it is assumed to be the result of microcirculatory alterations initiated during CPB and the vasospasm of mesenteric artery, its pathomechanism is as yet unclear. It is considered that it is caused by not only the invasion with cardiovascular surgery itself but also various factors such as the exacerbation of general conditions and using each therapeutic medication for it after surgery [8, 9]. In previous studies, its incidence rate was reported to be about 0.4 to 9.0%, and mortality was 30 to 93% [1–5]. In the present study, the incidence rate was 1.91% and in-hospital mortality was 75.0%, which was a similar result.

We have reviewed the clinical data of 12 NOMI cases and assumed the mechanism and the cause at the onset. Almost all the patients used high-dose NOE after surgery because of their condition of LOS, sepsis, or need for hemodialysis. When we investigated their clinical course in detail, it turned out that there were several factors that developed in combination at the onset of NOMI. In some cases, NOE was dose up just before the onset of NOMI due to the deterioration of LOS and sepsis or at the beginning of dehydration by hemodialysis. The effect of NOE, which induces vasoconstriction, increases resistance in peripheral splanchnic vessels, and stimulates β receptors in a dose-dependent manner to increase intestinal oxygen consumption, is likely to cause mesenteric ischemia [2, 5, 10]. As the result of the multivariate analysis of this study, the use of NOE > 0.10 μg/kg/min has shown significantly high OR and may have a strong association with the development of NOMI. It is difficult to detect the definite cause of NOMI because this cannot be explained by only the use of NOE and several factors may be related in complicated. However, the status of NOE and the presence of LOS, sepsis, and hemodialysis, which may be the reason for the use of NOE, can be the important factor in the pathogenic mechanism of NOMI.

Selective catheter angiography for mesenteric artery remains the gold standard for the diagnosis of NOMI. In previous studies, angiography was performed for all patients with suspected mesenteric ischemia because of the decreased intestinal peristalsis after cardiovascular surgery [2, 3]. In addition to the detection of the mesenteric arterial vasospasm, the inserted catheter allows the selective mesenteric intraarterial infusion of vasodilatative drugs. Using this diagnosis and treatment method, the result of low mortality was reported [3]. However, there were also reported cases that required surgical exploration and intestinal resection eventually, so its therapeutic effects are not clear yet [3, 11]. Angiography is an invasive procedure that cannot be applied for all patients with suspected symptoms.

In CT scan findings, pneumatosis intestinalis and portal venous gas are specific radiological signs, but both may be seen as nonischemic conditions [12]. Hasan et al. have reported that, among 26 patients with suspected NOMI from CT scan findings, 13 (50%) patients have confirmed bowel ischemia by surgical exploration and definite diagnosis of NOMI [7]. They have indicated the inaccuracy of the diagnosis by CT scan findings and necessity of surgical exploration without delay. A surgical exploration is only reliable way to provide an accurate assessment of bowel viability and necrotic sections requiring segmental intestinal resection [7, 10, 12]. Therefore, when we suspected the presence of intestinal ischemia from the comprehensive evaluation of the clinical course and laboratory data, we make it a rule to perform the CT scan and surgical exploration without hesitation.

In previous studies, the analysis for each laboratory data to predict and detect NOMI has been reported. In particular, there are several studies that indicate the elevation of lactate at the onset of NOMI [2, 3, 5, 13]. However, because hyperlactatemia can be found in various conditions, it is difficult to distinguish whether the cause of hyperlactatemia is NOMI or other factors [6, 14]. Simon et al. provided the results of analyzed laboratory data including lactate, creatine kinase, and lactate dehydrogenase isozyme. There were no significant differences between confirmed and negative diagnoses among patients with suspected NOMI [12].

In the present study, 7 of 12 cases were diagnosed with NOMI with hyperlactenemia as initial findings. However, because the elevation of serum lactate level was thought to be reflected irreversible and lethal mesenteric ischemia, all of 7 cases having shown hyperlactatemia could not be saved. When the serum lactate level has been elevated, it is highly likely to be too late for cure. Thus, lactate has lower usefulness for early diagnosis, and rather nonspecific gastrointestinal symptoms such as abdominal pain or vomiting may be more useful. Also, among the 12 cases in this study, 3 cases who survived were diagnosed early from subjective symptoms such as abdominal pain, not objective laboratory data. However, it was difficult to find subjective symptoms early because almost all NOMI patients were under sedation for mechanical ventilation. Such a situation is often seen at the onset of NOMI and may impede the early diagnosis.

Although there have been several studies that reported predictive factors for occlusive mesenteric ischemia after cardiovascular surgery or NOMI during intensive care, only a few focused on NOMI after cardiovascular surgery [2, 5, 13–19]. As the result of this analysis, NOE > 0.10 μg/kg/min and prolonged ventilation were identified as isolate risk factors for the onset of NOMI. The prolonged ventilation may be the cause of mesenteric ischemia because of peripheral hypoperfusion under sustained sedation and long-term bedridden condition [2, 15–18]. It is also suggested that PEEP decreases mesenteric blood flow and lung injury by mechanical ventilation can spread the pulmonary inflammation to distant organs [20, 21].

In this present study, we have further analyzed the use of NOE and prolonged ventilation as independent risk factors for NOMI, focusing on corresponding cases. This is because we considered that the definite cutoff value of NOE and ventilation factor can be further effective to predict the incidence of NOMI. For the NOE factor, total quantity, duration, and maximum dose of NOE were analyzed. For the prolonged ventilation factor, maximum PEEP, total ventilation time, and index calculated from PEEP * ventilation time/ BW were analyzed. However, between NOMI and non-NOMI groups corresponding to each factor, there were no significant differences and definite cutoff value could not be calculated in both factors **(**Table 5**)**. This is because each patient status after cardiovascular surgery was individually different, and the threshold of the onset of mesenteric ischemia was varied depending on the patient’s general conditions. For example, even between patients who used the same high dose of NOE, there are several factors related to intestinal blood flow. Consequently, the actual dose that can be the cause of mesenteric ischemia for each case is not expected to be the same. If insufficient circulatory dynamics or severe arteriosclerotic change is present, mesenteric ischemia can be caused by an even lower dose of NOE. The same applies to the ventilation factor. Hence, the use of high dose of NOE and prolonged ventilation are important for the onset of NOMI; however, it is very difficult to detect the definite and detailed cutoff value.

There are several limitations to our study. Because this is a retrospective and single-center database study, the reliability of each data is insufficient. In particular, suspecting the onset of NOMI and judging to perform the CT scan and laparotomy are dependent on the surgeon’s judgment. Furthermore, we have detected the independent risk factors from the multivariate regression model, but statistical reliability does not seem to be high because of a small number of NOMI cases.

There may be patients with mesenteric ischemia who were excluded from the NOMI group as we did not confirm mesenteric ischemia with surgical exploration. We confirmed no occlusion of mesenteric vessels from the CT scan findings instead of the selective mesenteric angiography. The cause of mesenteric ischemia may have been the occlusion or thrombosis.

## Conclusions

We have reviewed 12 cases who developed NOMI after cardiovascular surgery in our institution. Rapid prediction and diagnosis are essential to lower mortality. However, they are very difficult in practice because almost all patients with NOMI were under sedation and mechanical ventilated; thus, their prognosis was extremely poor.

In the multivariate logistic regression model, we have detected the use of NOE > 0.10 μg/kg/min and prolonged ventilation as independent risk factors of NOMI after cardiovascular surgery. In particular, we consider that the status of NOE and the presence of the factor, which can be the reason for the use of NOE, are strongly associated with the onset of NOMI. For the patient with suspected NOMI who have these risk factors, early CT scan and surgical exploration should be performed without delay.

## Abbreviations

AAR: Ascending aortic replacement
BW: Body weight
CPB: Cardiopulmonary bypass
CT: Computed tomography
IABP: Intraaortic balloon pump
LOS: Low output syndrome
MVP: Mitral valve plasty
MVR: Mitral valve replacement
NOE: Norepinephrine
NOMI: Nonocclusive mesenteric ischemia
OPCABG: Off-pump coronary artery bypass graft surgery
OR: Odds ratio
PCPS: Support and percutaneous cardiopulmonary support
SD: Standard deviation
TAR: Total arch replacement
